# Current status of cystic echinococcosis control in the Falkland Islands: has elimination been achieved?

**DOI:** 10.1017/S0031182023000100

**Published:** 2023-04

**Authors:** D. West, S. Pointing, H. S. Randhawa, A. Mastin, M. T. Rogan

**Affiliations:** 1 School of Science, Engineering & Environment, University of Salford, Salford M5 4WT, UK; 2 Department of Agriculture, Falkland Islands Government, Stanley FIQQ 1ZZ, Falkland Islands; 3 Fisheries Department, Falkland Islands Government, Stanley FIQQ 1ZZ, Falkland Islands; 4 Faculty of Life and Environmental Sciences, University of Iceland, 102 Reykjavík, Iceland

**Keywords:** Cestode, copro-antigen, copro-PCR, *Echinococcus*, hydatid, Taeniidae

## Abstract

Attempts to control cystic echinococcosis (CE) caused by *Echinococcus granulosus* in the Falkland Islands have been ongoing for over 50 years. No human cases have been recorded since the 1980s but there is a need to establish if the parasite has been completely eliminated from domestic animals. A study was carried out in 2018/2019 to identify dogs infected with *E. granulosus* using copro-antigen and copro-polymerase chain reaction (PCR) analysis. In addition, annual slaughter data were analysed to establish infection levels of *E. granulosus* and 2 other taeniid parasites. Results showed that 4 out of 589 dogs (0.7%) tested positive by copro-antigen analysis. Results from similar surveys carried out in 2010, 2012 and 2014 showed 17 (3%), 0 and 6 (1%) copro-antigen-positive dogs, respectively, with 8 dogs being confirmed by PCR in 2010. Annual abattoir data showed that from 2006 to 2020, 36 sheep were identified with *E. granulosus* (mean 0.0055%), 14 186 sheep with *Taenia hydatigena* (mean 2.2%) and 465 with *Taenia ovis* (mean 0.072%). Prevalences of *T. hydatigena* and *T. ovis* showed spontaneous rises in certain years where the infections could also be detected in lambs indicating that viable taeniid eggs were present. Observations of farm management procedures indicated that there were occasions when dogs could get access to infective taeniid material. In conclusion, *E. granulosus* is still present in sheep and dogs but at low prevalences. The increasing presence of *T. hydatigena* however, indicates that control measures are defective in some areas and there is potential for a re-emergence of CE.

## Graphical abstract

**Figure:**
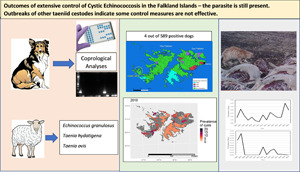

## Introduction

Cystic echinococcosis (CE) is caused by the cestode *Echinococcus granulosus* (*sensu lato*) and is one of the neglected tropical diseases which causes significant infection in many parts of the world. As a zoonosis, dogs and various ungulates are involved in the natural life cycle with humans becoming infected through accidental ingestion of eggs from dog feces contamination (Romig, 2003). The parasite exists as a complex of several genotypes, often infecting different intermediate hosts (*E. granulosus sensu lato*), with the G1 genotype (*E. granulosus sensu stricto*) occurring mainly in sheep being the most frequent genotype affecting humans (Alvarez Rojas *et al.*, 2014).

Since the mid-19th century, the public health importance of CE has been recognized and attempts to control its transmission have resulted in successful elimination being achieved in several island-based regions including Iceland (1863–1890), New Zealand (1959–2002) and Tasmania (1965–1996). In other non-island areas such as Argentina (Rio Negra) and Uruguay, a reduction in prevalences in humans, dogs and sheep have been achieved but without completely stopping transmission, even after several decades of effort (Larrieu and Zanini, 2012; Craig *et al.*, 2017). Successful control programmes have included 4 important components: (1) regulation of slaughter activity and disposal of offal; (2) prevention of dogs accessing offal; (3) de-worming of dogs (most frequently with praziquantel) and (4) public health education (Craig *et al*., 2017).

### CE in the Falkland Islands

The Falkland Islands (Malvinas) are an archipelago situated approximately 500 km off the east coast of Argentina, consisting of 2 main islands, West Falkland, and East Falkland, and 776 smaller islands with a total area of 12 000 km^2^. Agriculture in the Falklands makes up a large proportion of the economy, with the main income coming from the wool industry. There are 466 364 sheep on the Falkland Islands (according to 2020 farming statistics) farmed across 86 farms with a combined total of 1 129 723 hectares (Epstein *et al.*, 2007; Department of Agriculture, 2020). In 2003, the Sand Bay abattoir was opened. This is a European Union (EU)-registered abattoir, operating from early January to the end of May for the export of lamb, mutton and beef to the EU. In 2020, a total of 44 202 sheep were slaughtered. In addition, many farms also carry out some home slaughter of sheep for personal consumption, dog food or culling of old animals. In total, 51 farms were recorded as having dogs present and the total dog population in 2018–2019 was 589.

It is thought that *E. granulosus* was first introduced *via* live sheep imports from South America (Whitley, 1983). The first recorded case of CE discovered in the Falkland Islands was in 1941, when a single sheep out of a group of 2000 that were inspected was found to have a hydatid cyst (Gibbs, 1946). *Echinococcus granulosus* subsequently spread rapidly throughout the sheep and dog population, aided by the regular feeding of sheep offal to dogs and the lack of dog controls. The prevalence of infection in sheep peaked in 1969 when 59.3% of sheep slaughtered at the abattoir were found to be infected with CE (Reichel *et al.*, 1996). As the parasite prevalence in sheep increased, human cases were also identified. In 1965 CE was recognized as a public health risk and between 1965 and 1975, 11 human cases of CE were diagnosed within the small population of around 3000 people (Whitley, 1983). The Falkland Islands government (FIG) implemented the Tapeworm Eradication (Dogs) Order No. 1 (1965). This was the first legal action taken in the Falklands and involved regular purging of dogs using arecoline acetarsol (tenoban).

Subsequent modifications to the legislation were made between 1970 and 1981 (Whitley, 1983) finally resulting in the Hydatid Eradication (Dogs) Order 1981 (Falkland Island Government, 1981). This order combined and finalized previous orders relating to housing of dogs, disposal of offal and dosing of dogs with praziquantel (droncit/drontal) and is still in place today, with the only change coming in 2010 when the dosing schedule was reduced from 6 to 5 weeks to ensure the gap between dosing of dogs was significantly shorter than the prepatent time of *E. granulosus*.

### Outcomes of prolonged control programme in the Falkland Islands

The control programme in the Falkland Islands is considered to be successful as there has been no clinical confirmed human case since 1975 (Whitley, 1983) although 18 serological positive cases were identified in 1988 (Reichel *et al*., 1996). However, a single clinical case was confirmed in 2022 in an older woman but is likely to have been there for many years (Falkland Islands Chief Medical Officer, personal communication). The prevalence of CE in sheep at the abattoir declined from 47% in 1972 to 3% in 1982 (Whitley, 1983), with further slaughter data showing that the prevalence in sheep at abattoirs had fallen below 1%. However, from 1991 to 1993 there was an increase in prevalence from 0.11 to 0.47% (Reichel *et al*., 1996). Data are not available on the prevalence of *E. granulosus* in dogs prior to control measures being introduced and the only study investigating this was in 1996 where all dogs (*N* = 908) were serologically tested for anti-*E. granulosus* antibodies with 2.1% testing positive, and 1.7% testing positive by copro-antigen analysis, suggesting some dogs had access to hydatid cysts despite the control measures in place (Reichel *et al*., 1996). With over 50 years of control measures being implemented, there is a need to establish if complete elimination of the parasite from the Falkland Islands can be achieved. The objective of the current study was, therefore, to establish the current situation regarding *E. granulosus* infections in dogs and sheep and the prevalence of both *Taenia hydatigena* and *Taenia ovis* in sheep, as these parasites have a similar dog–sheep life cycle as *E. granulosus* (Gemmell *et al.*, 1986). In addition, conditions on individual farms in relation to CE control measures were examined to establish possible breakdown of control.

## Materials and methods

### Dog fecal samples

*Echinococcus granulosus* infections in dogs can be detected by both copro-antigen and copro-polymerase chain reaction (PCR) analysis. Both methods can differ in sensitivity and specificity with copro-PCR being more sensitive and specific when mature egg-laying adult worms are present, whilst copro-antigen testing is more robust and capable of detecting prepatent infections, but not low worm burdens (Craig *et al*., 2015). For copro-antigens, fresh fecal samples (3–5 g) were collected from individual dog kennels on each of the 51 farms which owned dogs (589 dogs in total), between 12th March 2018 and 27th April 2018. Samples were placed into a universal tube with 10 mL phosphate-buffered saline containing 0.3% Tween 20 and 10% formalin. Samples were homogenized with a wooden spatula, and then centrifuged at 2500 rpm (1125 ***g***) for 5 min. The supernatant was then removed and placed in 1.5 mL Eppendorf tubes and stored at −20°C until transfer to the University of Salford. Here, samples were stored at −80°C for 7 days to ensure that no viable eggs were present and then maintained at −20°C.

Control positive samples were obtained from purged dogs from a previous study in Kyrgyzstan (van Kesteren *et al*., 2013). Control negative samples were obtained from UK pet dogs and from post-mortem autopsied dogs from China (van Kesteren *et al*., 2015).

**Fig. 1.**
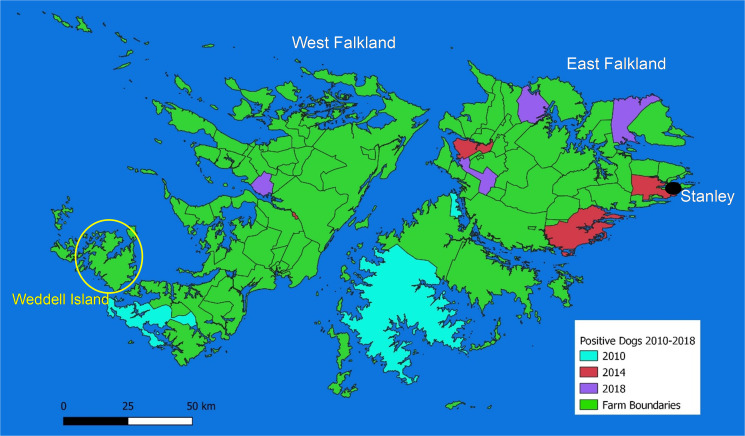
Locations of farms where dogs have tested copro-antigen positive between 2010 and 2018. The area around Weddell Island is where Patagonian foxes exist.

A population of Patagonian foxes (*Lycalopex griseus*) exists on 1 small group of remote islands to the west of West Falkland and 22 scat samples were collected from around fox dens in different locations on Weddell Island (Fig. 1) by the farmer and sent to the Department of Agriculture where they were analysed using the same protocols as for the copro-analysis of the domestic dog samples.

### Copro-antigen ELISA antibodies

Copro-antigen enzyme-linked immunosorbent assays (ELISAs) were carried out using a capture system involving purified rabbit immunoglobulin G antibodies produced in a previous study at the University of Salford by Dr Freya van Kesteren (2015). The specificity and sensitivity of this capture antibody system was tested using a panel of fecal samples from necropsied dogs in China (van Kesteren, 2015) and was found to be 93% sensitive as tested with 31 known positive samples and 100% specific using 43 known negative samples including 4 samples with *T. hydatigena* and 2 with *Taenia multiceps*. However, it is acknowledged that the number of control dogs used is relatively low and the likelihood of the copro-antigen test showing cross-reactivity with other taeniid cestodes may be as high as 30% (Hartnack *et al*., 2013).

A positive/negative cut-off value was estimated as the mean optical density (OD) value plus 3 standard deviations from a panel of 12 negative dogs from the Falkland Islands which had been previously tested by copro-PCR and copro-ELISA.

A previous copro-antigen survey of the Falkland Islands' dog population was also carried out in 2010, 2012 and 2014 (unpublished data).

### PCR analysis

Duplicate samples were also collected from each of the 589 dogs and stored in 90% ethanol prior to DNA extraction using a QIAamp^®^ Fast DNA Stool kit (Qiagen, Hilden, Germany) following the manufacturer's instructions. DNA from positive copro-ELISA samples was concentrated using ethanol precipitation. The PCR methodology for collection of *E. granulosus* fecal material was that described in Boufana *et al*. (2013) with some minor alterations in the PCR reaction mix. The primers follow the specific nucleotide sequence of the *E. granulosus* genotype 1 (G1) Nicotinamide adenine dinucleotide + hydrogen (NADH) dehydrogenase subunit 1 (ND1) mitochondrial gene: Eg1F81, 5′-GTT TTT GGC TGC CGC CAGAAC-3′ and Eg1R83, 5′-AAT TAA TGG AAA TAA TAACAA ACT TAA TCA ACA AT-3′ (Boufana *et al*., 2013). Universal cestode primers P60F, 5′-TTAA GATA TAT GTG GTA CAG GAT TAG ATA CCC-3′ and 5′-AAC CGA GGG TGA CGG GCG GTG TGT ACC-3′ (von Nickisch-Rosenegk *et al.*, 1999), targeting the mitochondrial 12S rDNA region, were used to detect other *Taenia* species, including *T. hydatigena*. Negative controls using PCR-grade water (Sigma-Aldrich, Dorset, UK) were used to identify contamination and positive controls from sequenced *E. granulosus* genomic DNA were used to ensure that the PCR reaction had worked. A previous copro-PCR survey of the Falkland Islands' dog population was also carried out in 2010 (unpublished).

### Prevalence of taeniid cestodes in sheep

At the abattoir, all animals are inspected by an EU-registered meat hygiene inspector as well as a vet from the Falkland Island Government Department of Agriculture. Meat inspection involves palpation of the liver and lungs to identify any cystic lesions. No slicing through organs takes place due to time constraints and it is, therefore, feasible that small cysts may go undetected. The infection status of each animal and the farm where it originated from is recorded and used to compile disease summary reports for each cull. Disease summary reports from 2007 to 2020 were used to compile a database for the current study. From 2011 onwards, the disease status of sheep passing through the abattoir was classified into age groups of new season lambs (NSL), yearling lambs (YL) and adult sheep. Lesions which were suspected of being *Echinococcus* cysts were removed and the contents examined under a binocular microscope for evidence of the presence of protoscoleces or the laminated layer of the cyst wall. From 2015 onwards, suspect material was also tested by PCR using *E. granulosus* G1 primers for the ND1 mitochondrial gene (von Nickisch-Rosenegk *et al*., 1999; Boufana *et al*., 2013). To confirm species and genotype, all positive PCR DNA was sequenced (Source Bioscience, Nottingham, UK) and analysed using a BLAST search of the NCBI GenBank database. The prevalences of other taeniid cestodes with a dog–sheep life cycle (*T. hydatigena* and *T. ovis*) were also recorded.

### Farming practice and *Echinococcus* control measures

In order to provide further understanding of the current management control measures of CE, visits, conversations and questionnaires were established with individual farms.

### Farm visits

Throughout the current study it was necessary to visit farms for the collection of dog fecal samples and other material. During these visits there was an opportunity to talk to farm owners/managers in an informal way and to observe the condition of dog kennels and offal disposal regimes.

### Questionnaires

A detailed questionnaire survey was carried out in 2018 to establish current farming practice relating to recommendations on dog management and offal disposal set by the FIG. Questionnaires were distributed by email prior to a visit only to farms which had registered dogs (51). Farms that did not own dogs were not visited or sent questionnaires.

### Local cull sites

Many farms cull a small number of sheep each year for personal consumption, dog food or because animals are too old for wool or meat production. Carcases of some of these animals are disposed of in remote areas of each farm and left on open ground and potentially made available to scavengers. To establish what was feeding on these carcases, 4 different farm sites were selected to erect camera traps (Bushnell Nature View CAMHD essential cameras) which were left in place for at least 2 weeks. The cameras were triggered *via* motion sensors and operated during both the day and night.

## Results

### Prevalence of *E. granulosus* in dogs

For the 2018 survey 589 fecal samples were tested for the presence of *E. granulosus* copro-antigen and 4 dogs from different farms were determined to be above the cut-off value of 0.21 (Fig. 1). All positive samples had considerably lower OD values for the 2 positive control samples, potentially suggesting low worm burdens or possible higher background activity than control negative dogs. The copro-ELISA was repeated to confirm positivity. However, none of these samples showed a positive PCR result using both *Echinococcus*-specific and generic taeniid primers. Dogs were, therefore, classified as suspect rather than confirmed infected. Previous unpublished surveys carried out in 2010, 2012 and 2014 showed 17 (3%), 0 (0%) and 6 (1%) copro-antigen positivity, respectively. For the 2010 samples 8 (1.4%) were also positive by PCR using *Echinococcus*-specific primers (unpublished data). PCR analysis was not carried out in the 2014 survey. None of the fox scats collected in 2018 gave a positive result. The location of farms where copro-antigen positive dogs were found is shown in Fig. 1.

Of the 9 farms which had copro-antigen positive dogs between 2010 and 2018, 7 had a history of hydatid cysts occurring in sheep and 5 had levels of *T. hydatigena* in sheep greater than the annual mean in both 2013 and 2019 (Table 1)

**Table 1.** Occurrence of *Echinococcus granulosus* and *Taenia hydatigena* in sheep from farms with copro-antigen positive dogs as identified in total dog population screening surveys in 2010, 2014 and 2018

Farm	Copro-positive dogs	Hydatid cysts present in sheep	*T. hydatigena* prevalence 2013 (%) (annual mean 2.4%)	*T. hydatigena* prevalence 2015 (%) (annual mean 3.2%)	*T. hydatigena* prevalence 2019 (%) (annual mean 6.0%)
EF22	2010	2010, 2014, 2015	4.30	**2.70**	6.00
WF15	2010	2018	7.20	4.10	8.90
EF1	2014	2008	3.10	**2.70**	n/d
EF5	2014	2019	**0.00**	**2.60**	8.70
EF7	2014	2016, 2018	6.80	5.80	7.70
WF23	2014	No	n/d	n/d	9.90
EF16	2018	2005	6.00	**1.60**	n/d
EF18	2018	No	7.80	**1.40**	6.60
EF23	2018	2020	3.20	14.80	7.00
WF18	2018	2020	9.30	7.80	8.40

WF, West Falkland; EF, East Falkland.

Bold values are years when the prevalence of *T. hydatigena* on the farm is less than the annual prevalence for the islands in total.

### Prevalence of *E. granulosus* and other taeniid infections in sheep passing through the abattoir

A total of 650 247 sheep from 86 farms passed through the single abattoir from 2006 to 2020, with a mean annual slaughter of 43 350 (range 30 029–57 798). Over this period, 36 sheep were identified with *E. granulosus* infection (mean 0.0055%, range 0.0000–0.0160%), 14 186 sheep were identified with *T. hydatigena* (mean 2.2%, range 0.5–6.0%) and 465 sheep were identified with *T. ovis* (mean 0.072%, range 0.000–0.450%). With *E. granulosus* all infections occurred in older sheep of commercial value, generally between 3 and 7 years of age, and were confirmed by microscopy and/or PCR. All of those tested by PCR belonged to the G1 genotype.

All 3 taeniid parasites have a similar dog–sheep cycle and Spearman's rank correlation coefficients were estimated to establish whether the prevalences of each species in sheep on individual farms were correlated. The prevalence of *E. granulosus* and *T. hydatigena* had a significant positive, though very weak correlation (*r*_s_ = 0.102, *P* = 0.026) in adult sheep but no correlation was observed between *E. granulosus* and *T. ovis* (*r*_s_ = 0.062, *P* = 0.179). A stronger, significant correlation was observed between *T. hydatigena* and *T. ovis* (*r*_s_ = 0.164, *P* ⩽ 0.001).

**Fig. 2.**
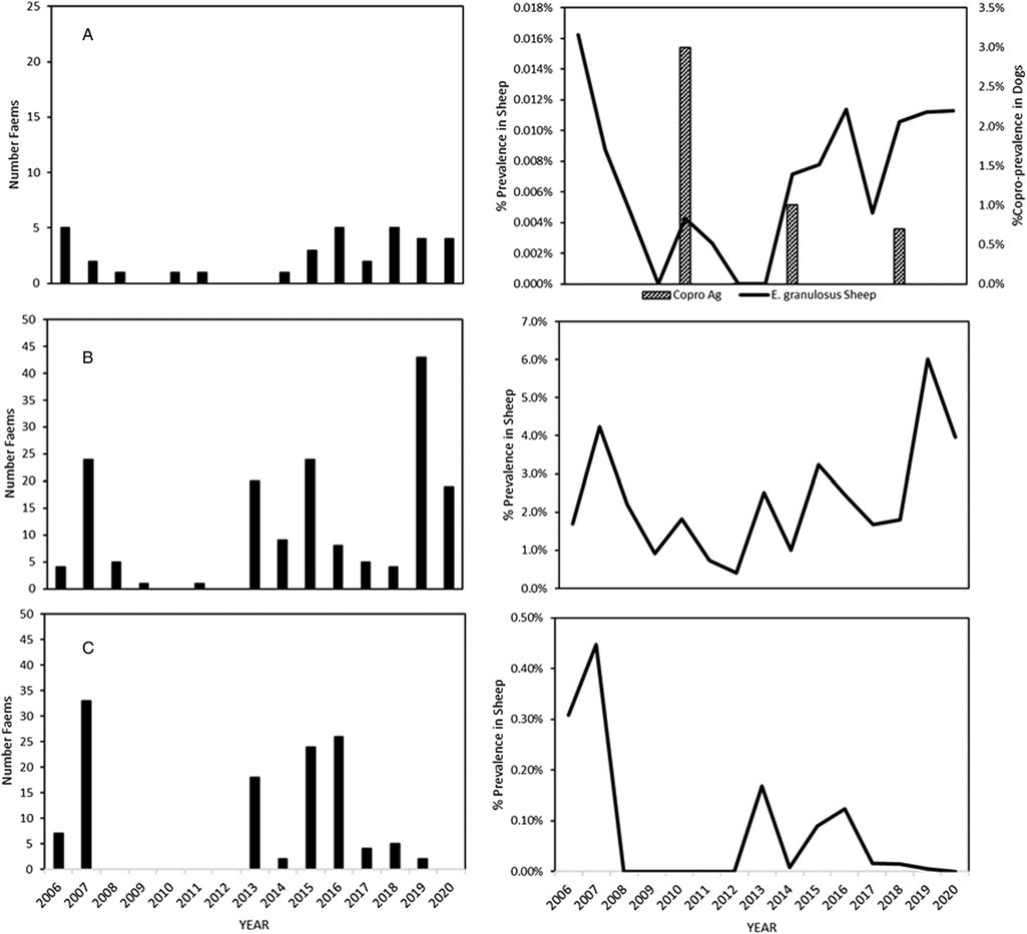
Prevalence of taeniid cestode larvae in sheep passing through the abattoir from 2006 to 2020: (a) number of farms with sheep having *Echinococcus granulosus* hydatid cysts, annual prevalence of *E. granulosus* in sheep across all farms (line) and prevalence of copro-antigen positive dogs (bar); (b) number of farms with prevalence of *Taenia hydatigena* >5% and annual prevalence *T. hydatigena* in sheep and (c) number of farms with prevalences of *Taenia ovis* and annual prevalence of *T. ovis* in sheep.

The prevalence of all 3 taeniid cestodes was greatest in 2006–2007 and *E. granulosus* was found in sheep from 5 different farms in 2007 (Fig. 2). Infection levels of all parasites in sheep dropped considerably after that until 2012, but subsequently rose again after 2013 with the exception of *T. ovis*, which declined again after 2016. No *E. granulosus* copro-antigen positive dogs were identified in 2012, but were identified in 2010 (17), 2014 (6) and 2018 (4).

**Fig. 3.**
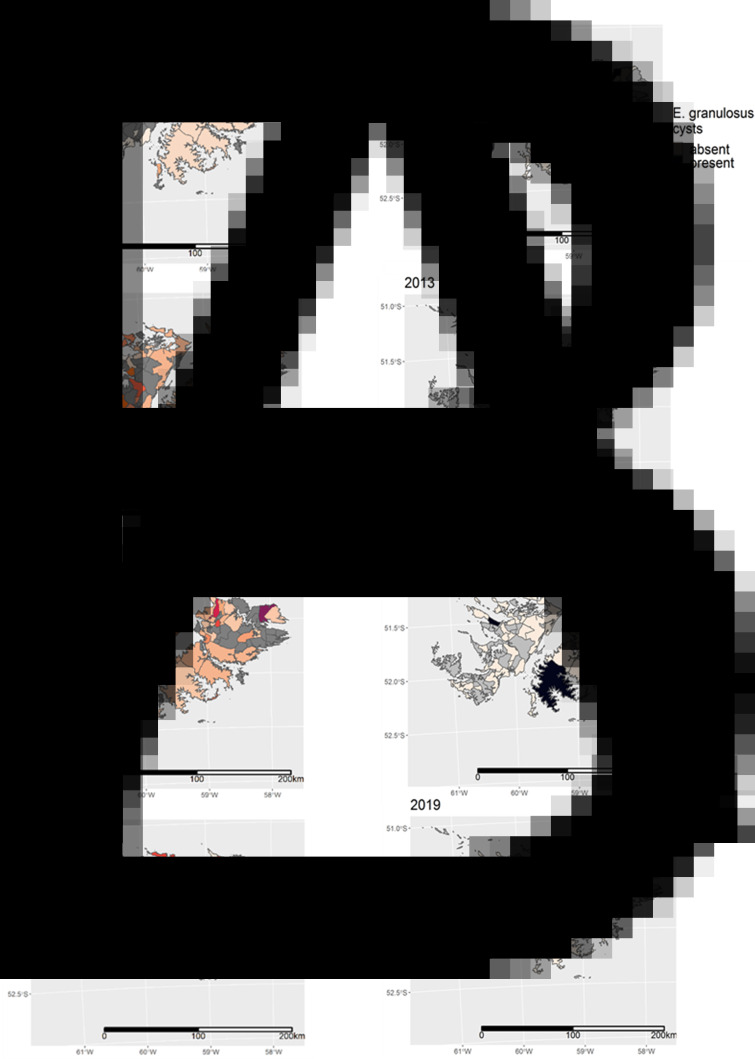
(a) Annual farm prevalence of *T. hydatigena* and (b) number and location of hydatid cysts in sheep identified between 2006 and 2020. In (a), coloured areas represent the prevalence of *T. hydatigena* on farms that provided sheep to the abattoir with grey areas representing farms that did not supply sheep to the abattoir. In (b), black areas represent farms where infected sheep are present, cream areas represent farms have no infected sheep and grey areas are farms that did not supply sheep to the abattoir.

The occurrence of *T. ovis* and *T. hydatigena* was more widespread than *E. granulosus* with *T. ovis* being present on 33 farms in 2007, 18 in 2013 and 26 in 2016, and *T. hydatigena* being present at some point on virtually all farms sending sheep to the abattoir (Fig. 2b). The number of farms with high prevalence of *T. hydatigena* (>5%) showed a similar distribution as the other parasites with peaks in 2007, 2013, 2015 and 2019 (Fig. 2b). The geographical distribution of farms having animals with *E. granulosus* did not show any obvious patterns to indicate ‘hot spots’ and there was considerable variation over time. For example, in 2006, 4 farms on West Falkland and 1 farm on East Falkland had *Echinococcus* infections whilst in 2019, 2 farms in West Falkland and 3 in East Falkland had infected sheep (Fig. 3). For *T. hydatigena*, geographical distribution maps showed that, in 2006, there were only 4 farms with prevalences >5% and 3 of these were in West Falkland, whilst in 2019, 43 farms had prevalences above this value and were spread in several areas across the islands (Fig. 3).

**Fig. 4.**
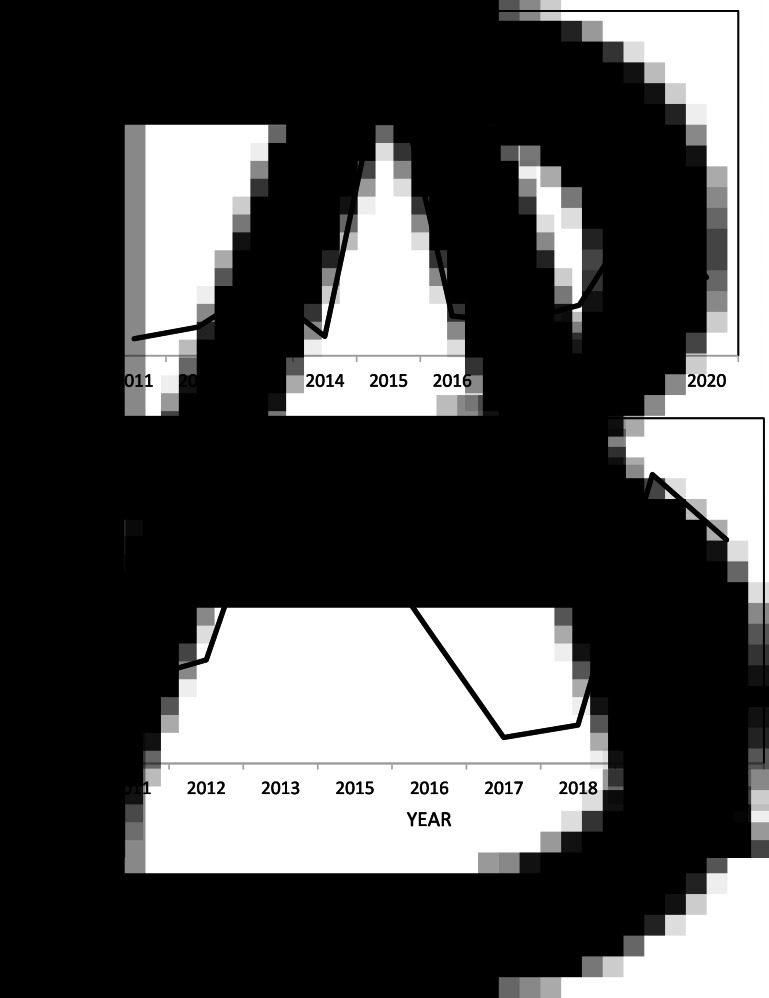
Prevalence of *T. hydatigena* in sheep from EF23 farm (a) and WF18 farm (b).

On individual farms, the presence of *T. hydatigena* in sheep often showed a rapid increase indicating the sudden presence of viable eggs in the environment, but the peaks of infection did not always occur at the same time. For instance, on EF23 farm (East Falkland), the occurrence in sheep rose rapidly from <1% in 2014 to almost 15% in 2015, whilst on WF18 farm (East Falkland), the initial peak of infection was in 2013 (Fig. 4).

**Fig. 5.**
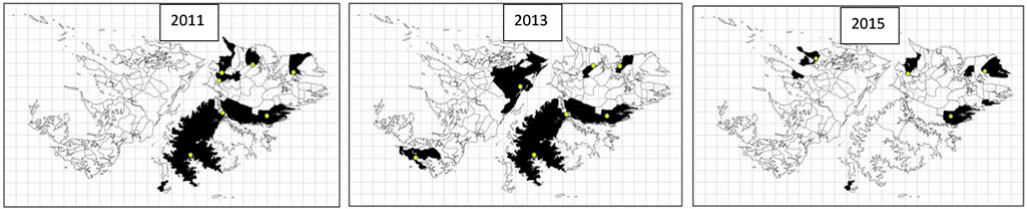
*Taenia hydatigena* in NSL in 2011 (left), 2013 (centre) and 2015 (right).

From 2011 onwards slaughter records included age classes of sheep. No *E. granulosus* infections were detected in any lambs. On farms where *T. hydatigena* occurred in NSL, prevalences were generally <3% but some farms occasionally had levels as high as 20%. The highest annual prevalences in NSL were between 2015 and 2018. In YL *T. hydatigena* was present as a mean annual prevalence of 1% across all years with the highest mean levels being in 2011, 2013 and 2020. *Taenia ovis* was only present in NSL in 2013 and in YL from 2013 to 2018. All infection levels were <1% on individual farms. Geographical distribution maps showed that in 2011, 10 farms recorded the presence of *T. hydatigena* in NSL and that all of these were on East Falkland (Fig. 5a), indicating that in that year, viable eggs were present in many areas of East Falkland. It should be stated however, that not all farms sent lambs to the abattoir. In 2013 6 farms on East Falkland and 5 farms on West Falkland showed the presence of infected NSL (Fig. 5b). In 2015 5 additional farms recorded the presence of *T. hydatigena* in NSL, 2 in West Falkland and 3 in East Falkland.

*Taenia ovis* infections were much less frequent and were only found in NSL in 2013 on 3 farms and in YL in 2013 and 2015 on 4 farms. The infection in adult sheep was present in 2007, but absent from 2008 to 2013 (Fig. 2c). Data from individual farms showed a similar trend to the overall data for the islands with peaks of infection occurring in adult sheep and NSL in 2013 with subsequent peaks in YL and adult sheep occurring in 2015 and 2016, respectively.

### Farm practice in relation to *Echinococcus* control

#### Dog management

**Fig. 6.**
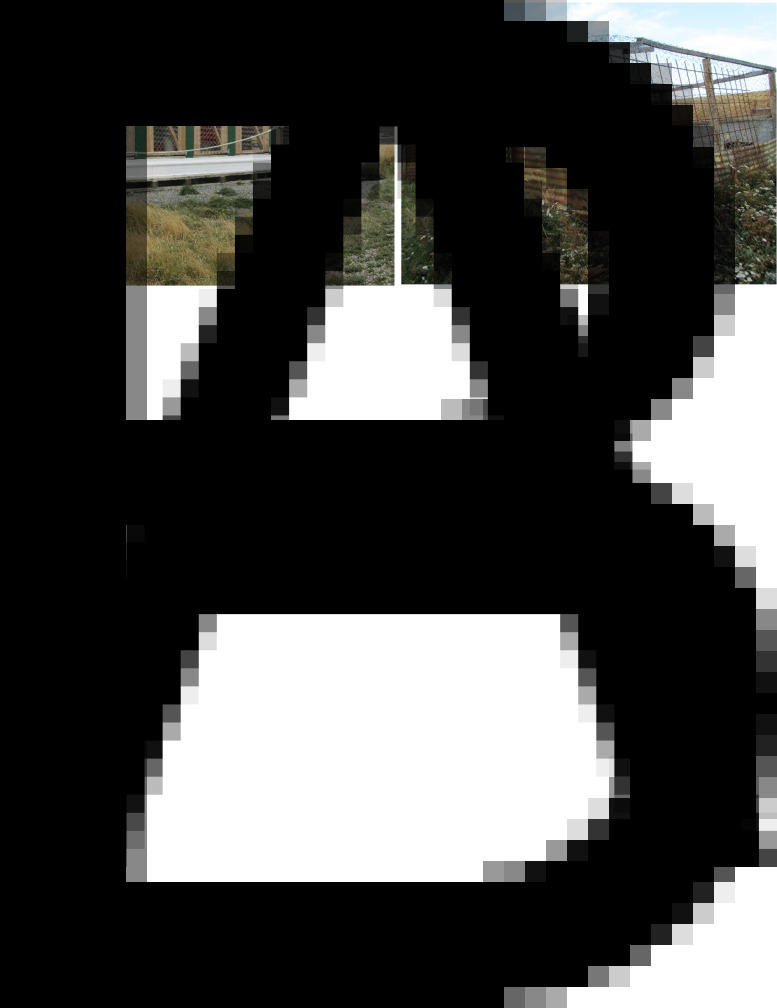
Example of dog kennels from different farms. Some (a) are well maintained whilst others (b) require maintenance.

In total 50 out of 81 farms were visited and 23 out of 51 (45%) questionnaires were returned from the surveyed farms, representing 49% of the working dog population on the Falklands (179 of 365). Dogs are legally required to be locked or tied up when not working and questionnaire results showed that 17 (74%) of the surveyed farms kept dogs locked in kennels when not working. Five farms (22%) kept dogs locked up by a combination of methods including tying up outside or keeping inside the home. One farm (4%) allowed dogs to roam freely on the property. The condition of kennels varied considerably with some being in excellent condition (Fig. 6a) and others in need of repair and upkeep (Fig. 6b). In total 96% of farms responding to the questionnaire fed raw meat to their dogs.

### Home slaughter of sheep and disposal of offal

Results indicated that all farms (100%) perform some home slaughter to provide meat for animal and human consumption or for disposal of old sheep, with the majority (78.3%, 18 of 23) also sending sheep to the abattoir for slaughter. Five farms (21.2%) only slaughtered at home and did not send any animals to the abattoir. Overall, approximately 3200 sheep are slaughtered annually at home across all the farms sampled with an average of 140 per farm (range 20–800). Three of the largest farms, that responded to the survey, killed between 200 and 800 animals per year. In relation to animals slaughtered for human or dog consumption, disposal of sheep plucks (liver, lungs and heart) was carried out on 19 farms (82.6%) by one of the designated procedures that would kill hydatid cysts, i.e. long-term storage in sealed containers, incineration or freezing before material was dumped on the shore or buried. However, 4 farms (19%) reported either dumping untreated offal directly on the shore or feeding it to pigs. It was noted that other offal such as intestines (potentially with *T. hydatigena* attached) was frequently dumped untreated on shorelines and get washed out to the sea.

**Fig. 7.**
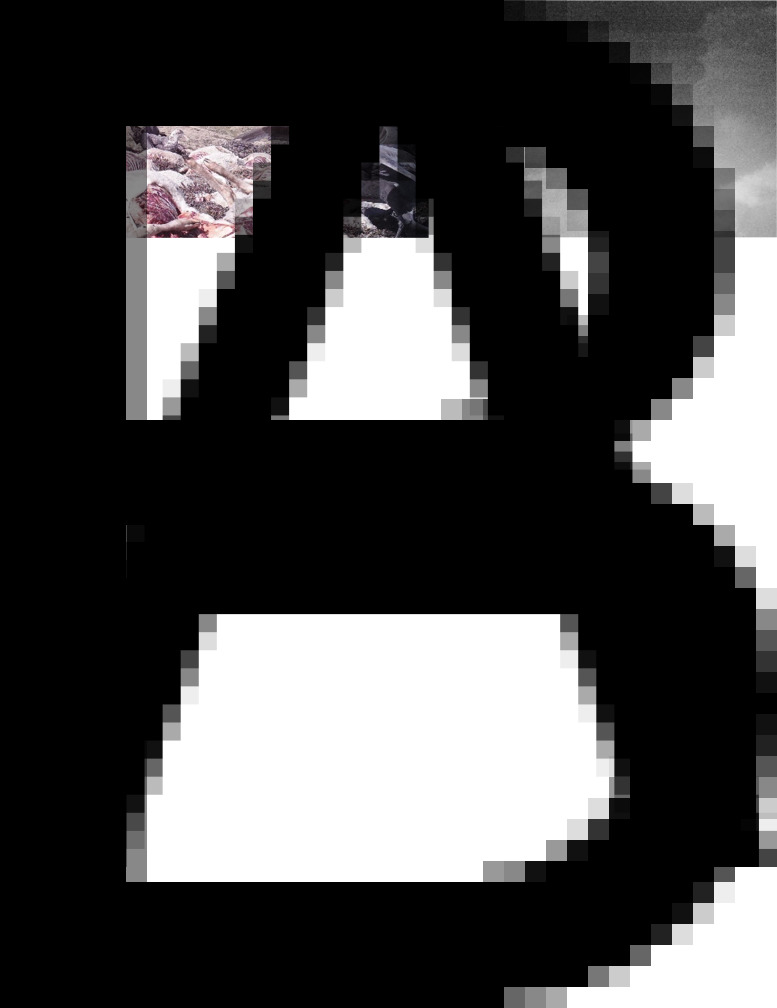
Camera trap images of a local cull site where sheep carcases are being scavenged upon by birds (a) and a dog (b).

In addition to sheep being slaughtered for human or animal consumption, many farms reported culling of older animals which were not of use for meat or wool production. This activity was carried out at distant sites away from the farm buildings and with culled animals simply being deposited on open ground or the shore with all internal organs intact. Camera trap photos showed that these animals were scavenged upon by feral cats and a range of birds, including Turkey vultures (*Cathartes aura*), crested caracara (*Caracara plancus*) and giant petrels (*Macronectes giganteus*) (Fig. 7a). On 1 occasion a single dog was observed feeding on sheep carcases at night (Fig. 7b). This farm had prevalences of *T. hydatigena* in sheep exceeding 5% on 4 occasions since 2011 (2013, 2015, 2019 and 2020) and recorded 2 sheep (0.4%) with hydatid cysts in 2020, and was also the location of one of the *E. granulosus* copro-antigen positive dogs in 2018.

## Discussion

The results of this survey clearly show that *E. granulosus* is still present in the Falkland Islands in sheep, but at very low prevalence with only 5 out of 43 976 sheep slaughtered in 2020 (0.0001%) having hydatid cysts. However, these data are only relevant to sheep passing through the central abattoir in Stanley and do not take into account any animals slaughtered on individual farms or at cull sites which may be considerably older and more likely to have a greater level of *Echinococcus* infection. With low prevalences the likelihood of transmission to dogs is considered to be very low, especially with the frequent dosing with praziquantel. However, the current study has shown that from 2010 to 2018, a small number of dogs were *E. granulosus* copro-antigen positive or copro-PCR positive using *E. granulosus*-specific primers. The fact that none of the 2018 dogs were PCR positive suggests that the copro-antigen test may be less specific and possibly reacting with other taeniid infections, possibly those which may be prepatent and not producing eggs (Hartneck *et al*., 2013; Craig *et al*., 2015). In addition, the amount of DNA in feces is very low and the copro-PCR technique is likely to underestimate the true prevalence. The additional presence of a relatively high number of sheep infected with *T. hydatigena* (1707 or 4% in 2020) however, provides evidence that some dogs are accessing sheep offal and are not being effectively dosed with praziquantel.

The possibility of other definitive hosts being involved in transmission is unlikely as the only other mammalian carnivores present are feral cats and Patagonian foxes. Whilst cats can occasionally act as competent hosts for *T. hydatigena* (Borji *et al*., 2011), but not for *T. ovis*, they are unlikely to produce the widespread distribution of infection observed in sheep (Figs 1, 3 and 4). Patagonian foxes are known definitive hosts for both *E. granulosus* and *T. hydatigena* in South America and are known to scavenge on carrion as part of their varied diet (Zanini *et al.*, 2006; Muñoz-Pedreros *et al*., 2018). However, the fox population on the Falklands is restricted to Weddell Island and a few smaller islands in the same area. Fox scat samples were negative for both copro-antigens and the presence of taeniid eggs. Previous investigations involving post-mortem dissection of Patagonian foxes found no evidence of taeniid infection (unpublished data). The contribution of foxes on Weddell Island to infection of sheep on East and West Falklands seems unlikely even though taeniid eggs can be transferred some distance by wind and insect vectors (Lawson and Gemmell, 1990; Torgerson *et al.*, 1995; Benelli *et al.*, 2021), as the distribution of infection in sheep is not concentrated in the farms closest to Weddell Island.

The distribution of *T. hydatigena* and *T. ovis* infections is strongly suggestive of the occurrence of a number of outbreaks particularly relating to high infections in 2007, 2013, 2015 and 2019 whereby the presence of parasites in adult sheep rises sharply. The occurrence of these parasites in lambs indicates the presence of viable eggs in the environment in that year. Similar situations have been reported in relation to ‘cysticercosis storms’ which occur whenever the general level of taeniid eggs in the environment is low and density-dependent constraints resulting from acquired immunity are not present in sheep (Gemmell *et al.*, 1990; Eichenberger *et al.*, 2011). The subsequent introduction of 1 or more infected dogs results in a rapid increase of infection in sheep.

Both *Taenia* parasites have a considerably greater biotic potential than *E. granulosus*, producing many more eggs for longer periods of time (Gemmell *et al.*, 1987). From the current data it is not possible to say where or when the dogs releasing eggs were located as taeniid eggs are known to survive for several years in suitable environments (Thevenet *et al.*, 2020; Jansen *et al*., 2021) and be transferred over great distances by wind and animal vectors (Jansen *et al*., 2021). However, the geographical distribution of infection, particularly of *T. hydatigena* is extensive indicating the probable presence of infected dogs on farms in more than 1 location. Infection of NSL in 2020 indicates that viable eggs are still present and that infected dogs must have been present in recent years. Modelling studies have shown that, because of the greater biotic potential and number of eggs produced, *T. hydatigena* and *T. ovis* are much more difficult to control and will persist in the environment for considerably longer than *E. granulosus* (Gemmell *et al*., 1986).

In surveying the practices relating to dog housing and disposal of sheep offal on individual farms, there were a number of points of high-risk concern. Whilst many farms disposed of sheep livers and lungs effectively using recommended methods, a small number of farms disposed of offal by leaving it untreated on shorelines to get washed out to the sea or fed directly to pigs. In both situations there is the potential for dogs to scavenge on this material and become infected. Dumping of sheep intestines on shores is also common practice on several farms and since *T. hydatigena* is frequently found in the intestinal mesenteries, this is a potential source of dog infection. A more worrying situation exists in relation to the culling of old sheep as this often involves carcases being dumped at specific sites some distance from many farms. This is perceived by some farmers as acceptable since dogs are not permitted to roam freely and would, therefore, not be able to access these carcases. However, the current study has shown that, in some cases, kennels used to house dogs are in poor condition and that, in at least 1 case, dogs were observed feeding on carcases at cull sites at night. The presence of many avian scavengers at these sites may lead also to the distribution of possible infective material over greater distances. In terms of future policy making to improve control, it is essential that this practice should cease since it has been shown that over 80% of the *Echinococcus* parasite burden in sheep is found in animals over 4 years of age (Torgerson *et al*., 2009).

As well as the potential for some dogs to become infected, the presence of significant levels of taeniid eggs in the environment must indicate that adult worms are persisting for some time beyond patency and the effectiveness of praziquantel dosing every 5 weeks is therefore not 100%. Parasite resistance to praziquantel is possible as this has been reported in relation to human schistosomiasis (Cioli and Pica-Mattoccia, 2003). However, drug-resistant strains have not been reported for any taeniid cestodes. It is known, however, that some taeniid worms do not respond to the drug and its overall effectiveness is around 95% (Gemmell *et al.*, 1977; Miro *et al.*, 2007). The more likely scenario is incorrect administration of the drug to dogs, either by not dosing, underdosing or dogs regurgitating tablets. The use of praziquantel in dogs has been associated with some problems such as its unpleasant taste and smell for dogs, inadequate estimation of dog's weight for proper dosing (underdosage) and reluctance of some owners to administer a large number of pills at each time deworming is required (Larrieu and Zanini, 2012, Larrieu *et al*., 2019). The possibility of environmental contamination of eggs arising from infected dogs in more distant endemic areas, such as Patagonia, is an interesting concept. It is known that taeniid eggs can be carried more than 50 miles (e.g. from mainland Scotland to St Kilda) (Torgerson *et al*., 1995) but distances of more than 300 miles may be too excessive. This idea would need further investigation by DNA sequencing analysis of different geographical isolates.

The *Echinococcus* control programme on the Falkland Islands has been in an ‘attack phase’ (Gemmell *et al*., 2001; Larrieu and Zanini, 2012; Craig *et al*., 2017) involving frequent deworming of dogs, restriction on dog movements, constraints on offal disposal and farmer education for more than 50 years and the parasite still persists, yet at very low prevalence in sheep. This time span is not dissimilar to New Zealand and Tasmania where elimination was declared after 43 and 31 years, respectively (Craig *et al*., 2017). Under such conditions it is likely that the parasite could be eliminated in the Falkland Islands in the near future and the very low prevalences of all taeniids from 2008 to 2012 is indicative of this. However, the sporadic occurrence of a small number of infected dogs and the rising levels of *T. hydatigena* in sheep on some farms are of significant concern that in some areas, control strategies may be breaking down. As such, it may be more effective to move into a ‘consolidation phase’ involving a more targeted approach and focusing on farms with a previous history of infected dogs or high *T. hydatigena* infection in sheep, with an emphasis on praziquantel administration under veterinary supervision and maintenance of restricted dog movement. Diagnostic techniques for dogs should involve screening for generic taeniid infection initially followed by copro-PCR confirmation of species. In addition, cessation of the dumping of sheep carcases at specific cull sites and disposal of offal on sea shores would significantly reduce the opportunity for dogs to be infected with all 3 taeniid cestodes.

## Data Availability

The datasets generated during and/or analysed during the current study are not publicly available due to Government ownership but are available from the corresponding author on reasonable request.
